# The Role of Fibronectin in the Adherence and Inflammatory Response Induced by Enteroaggregative *Escherichia coli* on Epithelial Cells

**DOI:** 10.3389/fcimb.2016.00166

**Published:** 2016-12-08

**Authors:** Dominique Yáñez, Mariana Izquierdo, Fernando Ruiz-Perez, James P. Nataro, Jorge A. Girón, Roberto M. Vidal, Mauricio J. Farfan

**Affiliations:** ^1^Centro de Estudios Moleculares, Departamento de Pediatría, Hospital Dr. Luis Calvo Mackenna, Facultad de Medicina, Universidad de ChileSantiago, Chile; ^2^Department of Pediatrics, University of Virginia School of MedicineCharlottesville, VA, USA; ^3^Programa de Microbiología, Instituto de Ciencias Biomédicas, Facultad de Medicina, Universidad de ChileSantiago, Chile

**Keywords:** enteroaggregative *E. coli*, adherence aggregative fimbriae, fibronectin, IL-8

## Abstract

Enteroaggregative *Escherichia coli* (EAEC) infections are still one of the most important etiologic pathogens of diarrhea in children worldwide. EAEC pathogenesis comprises three stages: adherence and colonization, production of toxins, and diarrhea followed by inflammation. Previous studies have demonstrated that EAEC strains have the ability to bind to fibronectin (FN); however, the role this extracellular matrix protein plays in the inflammatory response induced by EAEC remains unknown. In this study, we postulated that FN-mediated adherence of EAEC strains to epithelial cells increases the expression of pro-inflammatory genes. To verify this hypothesis, we infected HEp-2 and HT-29 cells, in both the presence and absence of FN, with EAEC reference strain 042. We quantified IL-8 secretion and the relative expression of a set of genes regulated by the NF-κB pathway. Although FN increased EAEC adherence, no changes in IL-8 protein secretion or *IL8* gene expression were observed. Similar observations were found in HEp-2 cells transfected with FN-siRNA and infected with EAEC. To evaluate the involvement of AAF/II fimbriae, we infected HEp-2 and HT-29 cells, in both the presence and absence of FN, with an EAEC 042*aafA* mutant strain transformed with a plasmid harboring the native *aafA* gene with a site-directed mutation in Lys72 residue (K72A and K72R strains). No changes in IL-8 secretion were observed. Finally, SEM immunogold assay of cells incubated with FN and infected with EAEC revealed that AAF fimbriae can bind to cells either directly or mediated by FN. Our data suggests that FN participates in AAF/II fimbriae-mediated adherence of EAEC to epithelial cells, but not in the inflammatory response of cells infected by this pathogen.

## Introduction

Enteroaggregative *Escherichia coli* (EAEC) are a major cause of acute diarrhea in developing and industrialized regions (Huang et al., 2006). Several outbreaks, such as the 2011 outbreak caused by a Shiga toxin-producing EAEC in Germany, have increased interest in investigating the molecular mechanisms involved in the interaction between this pathogen and the intestinal epithelia (Rasko et al., 2011). In general, EAEC pathogenesis involves adherence to intestinal cells, the secretion of toxins and the induction of an inflammatory response (Harrington et al., 2006). EAEC is characterized by its ability to adhere to HEp-2 cells in a “stacked-brick” aggregative adherence pattern. Clinically, EAEC induces watery diarrhea, sometimes accompanied by blood and mucus, and patients normally manifest intestinal inflammation (Steiner et al., 1998; Flores and Okhuysen, 2009).

It has been proposed that extracellular matrix (ECM) proteins act as receptors for several pathogens, including EAEC (Farfan et al., 2008). Under normal conditions, ECM proteins are restricted to the basement membrane, where they are not available for interaction with luminal bacteria (Dubreuil et al., 2002). However, several observations indicate that ECM proteins could be present on the surface of intestinal cells and may participate as bacterial receptors (Westerlund and Korhonen, 1993; Walia et al., 2004; Konkel et al., 2005). Fibronectin (FN) was the first ECM protein shown to act as a cell receptor for bacterial pathogens (Kuusela, 1978). This glycoprotein contains multiple domains that can bind to several ligands, such as fibrin, heparin, syndecan, collagens, gelatin, and integrins (Pankov and Yamada, 2002). To date, over 100 bacterial FN binding proteins have been identified, and several studies have shown that adhesion to FN would contribute to the successful colonization of bacteria on the epithelial surface, by acting as a “molecular bridge” connecting the bacteria with the host cell surface (Henderson et al., 2011; Izquierdo et al., 2014a).

EAEC adherence to intestinal cells is mediated by fimbrial adhesins, designated aggregative adherence fimbriae (AAFs). For EAEC reference strain 042, adherence to cells requires the AAF/II variant (Czeczulin et al., 1997), but the cell receptors involved in the recognition of these fimbriae have not been completely described. Our group, along with others, has previously shown the recognition of FN by AAF/II fimbriae, resulting in enhanced bacterial adherence to intestinal epithelial cells (Farfan et al., 2008; Konar et al., 2012; Izquierdo et al., 2014a).

Given the above, in this study we investigated whether the presence of FN increases the adherence of EAEC strains to epithelial cells, induces secretion of IL-8 and the expression of pro-inflammatory genes through the fimbriae. Our data suggest that FN increases AAF/II fimbriae-mediated adherence of EAEC to epithelial cells; however, in the case of cells infected with this pathogen, binding to FN is not involved in their inflammatory response.

## Materials and methods

### Bacterial strains

EAEC strains were grown overnight in Luria-Bertani (LB) broth. EAEC 042*aafA* mutant strains transformed with the pBAD30 plasmid harboring the native *aafA* gene with site-directed mutation in Lys72 (K72R and K72A) were selectively grown on LB-agar with kanamycin and 0.2% arabinose (Berry et al., 2014).

### Cell culture

HT-29 and HEp-2 cells were maintained in McCoy's 5A Medium (Gibco) and DMEM high glucose (DMEM/HG) (HyClone), respectively. Cells media were supplemented with 10% fetal bovine serum (FBS), penicillin (10 U/mL), and streptomycin (10 g/mL) at 37°C under 5% CO_2_.

### Adherence assays

Confluent epithelial cells were incubated for 30 min with FN (2.5, 5, 7.5, or 10 μg/well) and then infected with a multiplicity of infection (MOI) of 10 for 3 h, as previously described (Izquierdo et al., 2014b). Cells were lysed with 0.1% Triton X-100/PBS and serial dilutions of lysed cells were plated onto LB agar. The number of adherent bacteria was determined by counting colony-forming units (CFU).

### IL-8 secretion

Infection assay was performed with HEp-2 and HT-29 cells in triplicate, as described above. After infection, cells were incubated with media containing gentamicin (100 μg/ml) for 3 h to determine the amount of IL-8 by ELISA, as previously described (Harrington et al., 2005).

### siRNA

Subconfluent cultures of HEp-2 cells were transfected with 20 pmol of small interfering RNA (siRNA) for FN or scrambled siRNA (Santa Cruz Biotechnology), as previously described (Izquierdo et al., 2014b). Adherence and IL-8 secretion assays were performed 48–72 h after transfection. Reduction in FN expression was determined by Western blot analysis of transfected cells lysates using primary antibodies against FN (Sigma) or actin, and the secondary HRP-conjugated antibody. The band intensity was determined with ImageJ software. Additionally, transfected HEp-2 cells grown in an 8-well Lab-Tek Chamber slide were fixed and immunostained with anti-FN primary antibodies. FN expression was examined by confocal microscopy.

### Real-time PCR

RNA obtained from HEp-2 cells infected with EAEC was reverse-transcribed into single-strands using the Transcriptor First Strand cDNA Synthesis Kit (Roche). cDNA was used as a template to quantify the expression of *IL8, CCL20, TNFA, NFkB, IL1B*, and *FOS* genes using specific primers (Vergara et al., 2015). The mRNA expression levels were normalized to those of the human housekeeping gene glyceraldehyde 3-phosphate dehydrogenase (GAPDH), as previously described (Vergara et al., 2015), and analyzed using the comparative critical threshold (CT) method.

### Scanning electron microscopy

HEp-2 cells were infected for adherence assays, as described above. The wells were rinsed twice with PBS and fixed in 2.5% glutaraldehyde in PBS. A 1:50 dilution of immune rabbit serum against AafA was added for 1 h followed by washing and addition of the anti-rabbit IgG 10-nm gold conjugate (BBI solutions) (1:100) for 1 h. FN was detected with mouse anti-FN monoclonal antibodies (1:50) (Sigma) and goat anti-mouse Ig 20-nm gold conjugate (BBI solutions) (1:100) for 1 h each. The specimens were submitted to the Advanced Microscopy Facility (University of Virginia) for preparation; namely, post-fixed with osmium tetroxide, dehydrated in ethanol, critical point dried, mounted, coated with carbon, and imaged at 3 kV with a Zeiss Sigma HDS scope with SE2 and back scatter detectors to visualize the gold particles.

### Statistical analysis

Experimental data were expressed as mean ± SD in each group. Statistical comparisons were performed using the Student's *t*-test or ANOVA multiple pairwise comparisons test with Tukey's posttest. The significant *P*-value was established at *P* < 0.05.

## Results

### FN increases EAEC adherence to epithelial cells, but not IL-8 secretion

**Figure 1:**
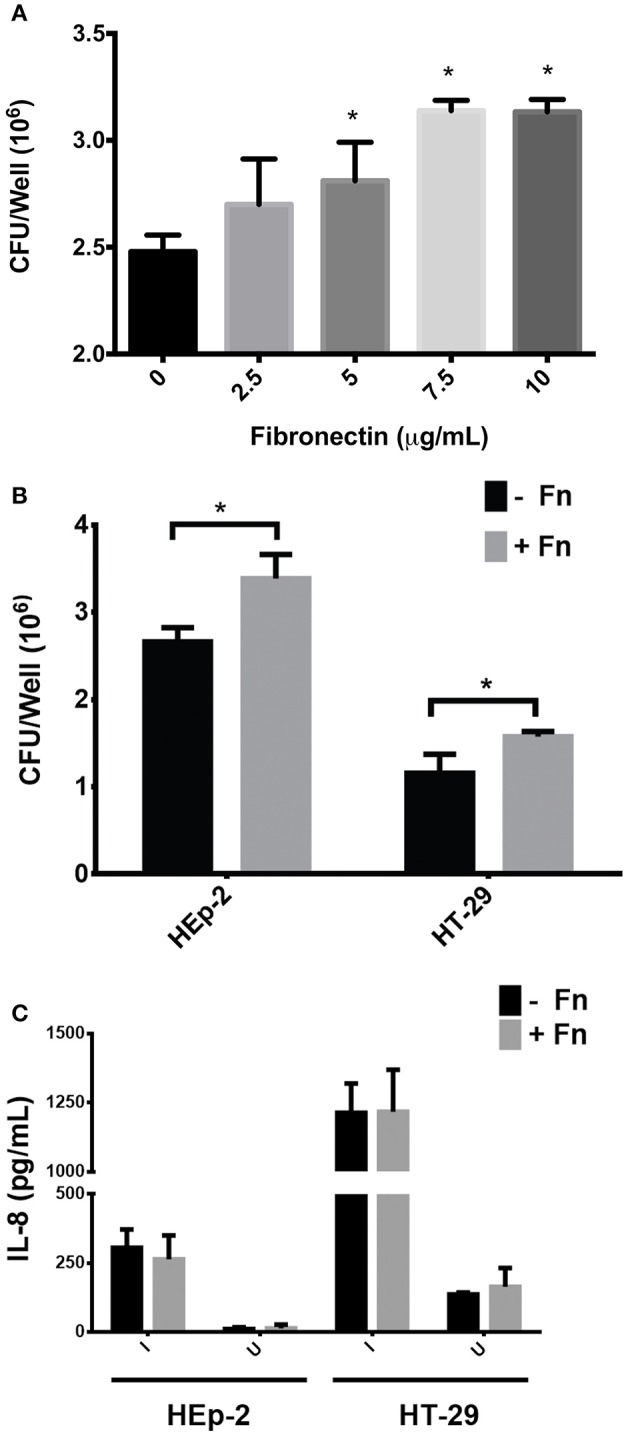
**Participation of fibronectin in adherence and IL-8 secretion (A)**. HEp-2 cells were pre-incubated with increasing concentrations of fibronectin prior to infection. The number of bound bacteria was determined by plating. **(B)** Prior to infection, HEp-2 and HT-29 cells were pre-incubated with 7.5 μg/mL of FN (+Fn) or media only (−Fn) and then infected with EAEC 042 strains for 3 h. The number of bound bacteria was determined by plating. **(C)** After 3 h of infection, cell monolayers were incubated with gentamicin for 3 h and the media from cells were collected. Secreted IL-8 levels were measured by ELISA. I, infected; U, uninfected. Bars represent the mean of three experiments, with the error bars indicating one standard deviation. ^*^Represents a statistically significant difference (*P* < 0.05).

HEp-2 cells were incubated with increasing concentrations of FN prior to infection with EAEC. Figure 1A shows that EAEC adherence to cultured epithelial cells increased in a dose-dependent manner. Using 7.5 μg/ml of FN, we evaluated the amount of IL-8 secreted to the media by HEp-2 and HT-29 cells 3 h post-infection with EAEC. Although there was a significant increase in EAEC adherence to both cell types (Figure 1B), no difference was observed in the amount of secreted IL-8 present in the media (Figure 1C). Thus, these data indicate that EAEC-induced IL-8 secretion in HT-29 and HEp-2 cells is FN independent. There was no observed effect of FN on the induction of IL-8 secretion in HEp-2 and HT-29 cells.

### FN-mediated adherence of EAEC to epithelial cells is associated with no changes in the expression of the *IL8* gene

**Figure 2:**
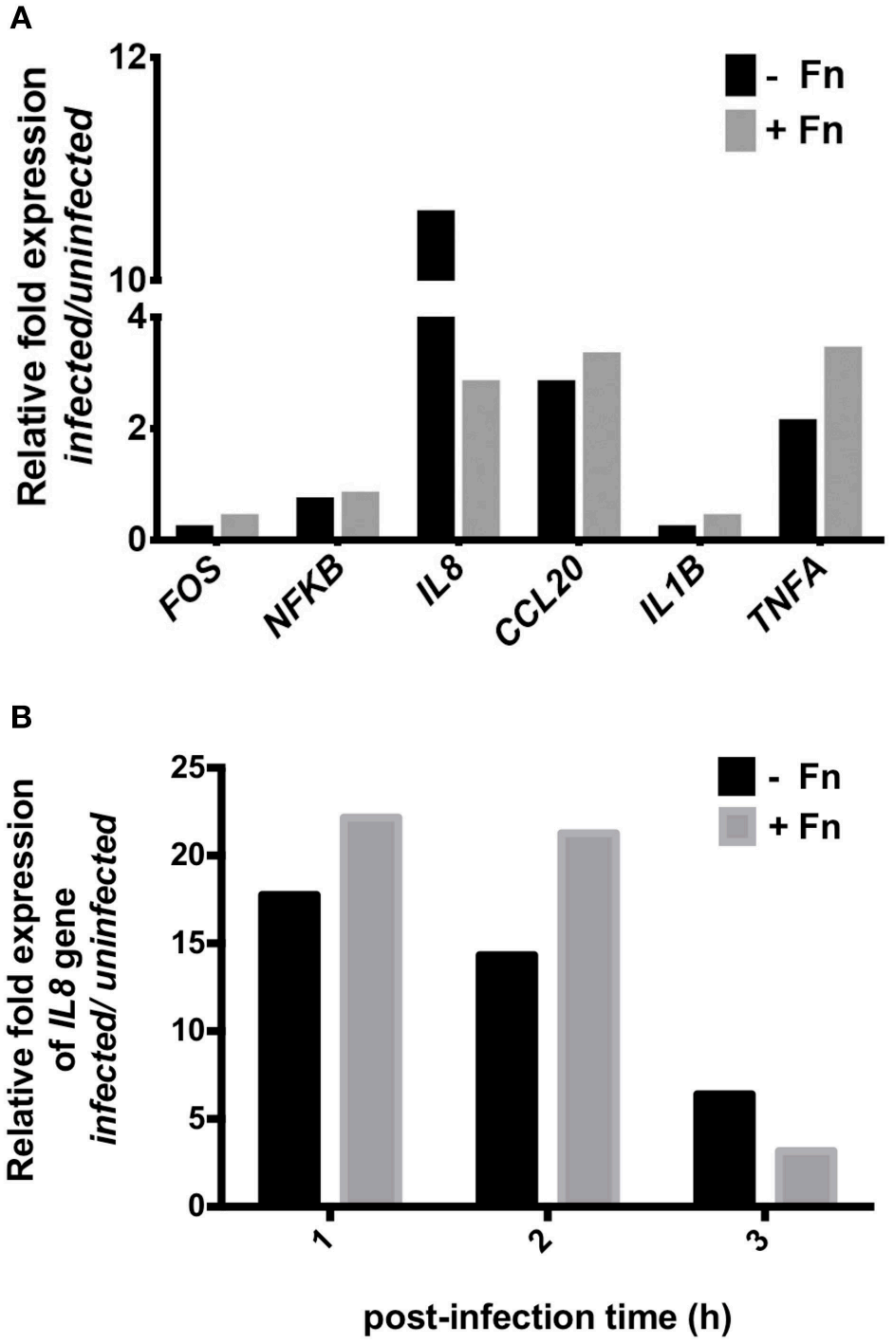
**NF-κB pathway gene expression in cells infected with EAEC in the presence of fibronectin**. Prior to infection, HEp-2 cells were pre-incubated with 7.5 μg/mL of fibronectin (+Fn) or media only (−Fn). **(A)** Real-time PCR analysis for *FOS, NFKB, IL8, CCL20, IL1B*, and *TNFA* genes after 3 h of infection. **(B)** Real-time PCR analysis for *IL8* gene at 1, 2, and 3 h post-infection.

To corroborate the results described above, we evaluated the expression of several pro-inflammatory genes associated with enteric infection. HEp-2 cells were infected with EAEC strain 042 in the presence or absence of FN and the relative expression of *FOS, NFkB, IL8, CCL20, IL1B*, and *TNFa* genes was compared to uninfected cells. After 3 h of infection, there was no difference in the expression of any of the pro-inflammatory markers; however, an ~4 fold reduction in the expression of the *IL8* gene was found in cells preincubated with FN as compared to uninfected cells (Figure 2A). Additionally, we performed the same analyses on cells 1, 2, and 3 h post-infection, and found no significant difference in *IL8* gene expression (Figure 2B).

### FN gene knockdown reduced EAEC adherence to epithelial cells, but had no effect on IL-8 secretion

**Figure 3:**
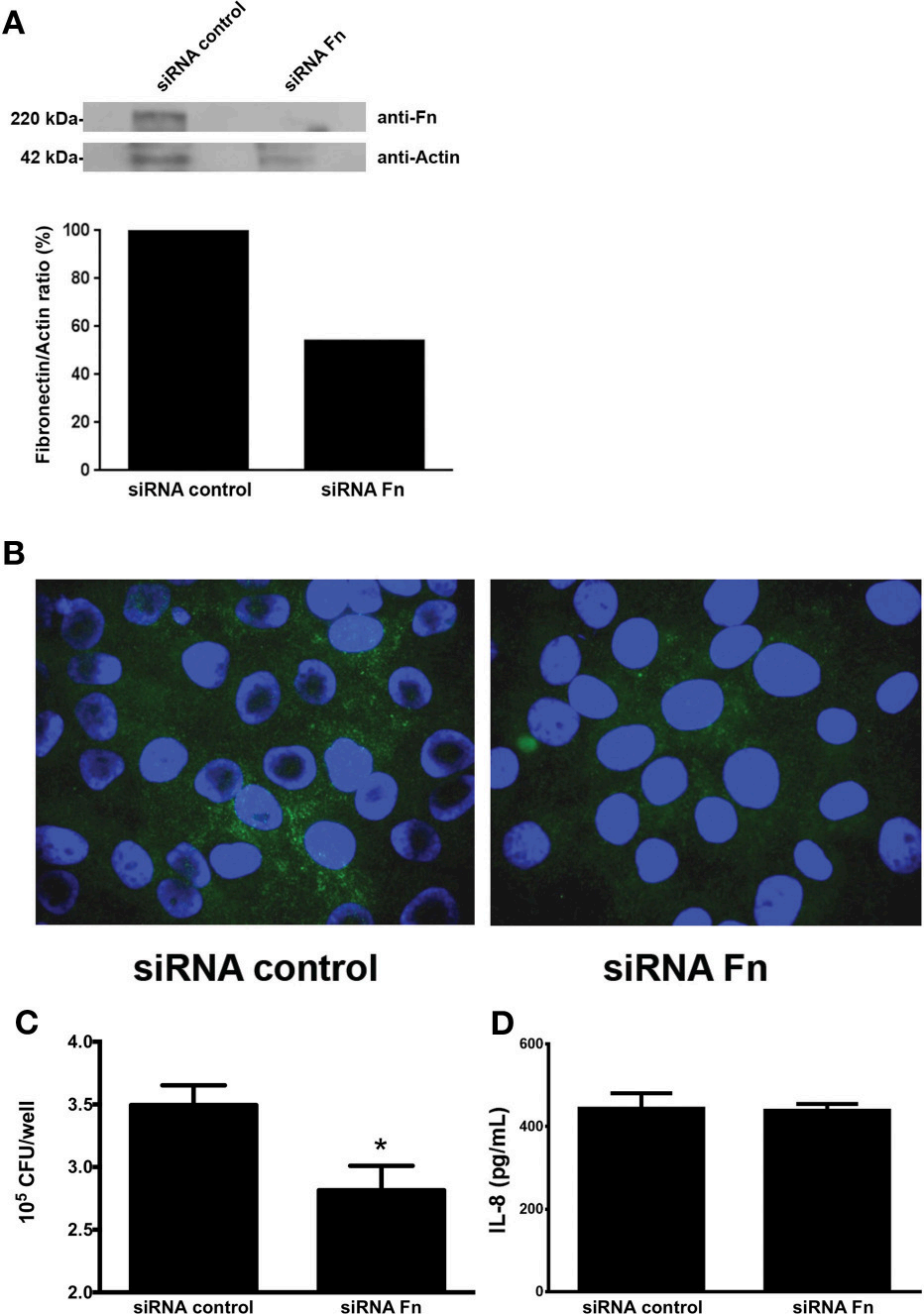
**Knocking down the fibronectin gene reduces EAEC binding to epithelial cells. (A)** Fibronectin expression determined by Western blot analysis using HEp-2 cells lysates transfected with fibronectin or scrambled siRNA (siRNA control). **(B)** Immunofluorescence of transfected HEp-2 cells infected with EAEC 042. Fibronectin was visualized with Alexa Fluor 488 (green), and bacterial or human DNA was stained with TO-PRO-3 (blue). **(C)** HEp-2 cells treated with siRNA infected with EAEC 042. The number of adherent bacteria was determined by counting CFUs. **(D)** Levels of secreted IL-8 at 3 h post infection. Bars represent the mean of three experiments, with the error bars indicating one standard deviation. ^*^Denotes statistically significant difference (*P* < 0.05).

The involvement of cell-produced FN in EAEC adherence and IL-8 secretion was evaluated in HEp-2 cells transfected with siRNA for FN. Reduction in FN expression, compared with HEp-2 transfected with scrambled siRNA, was confirmed by Western blot (Figure 3A) and immunofluorescence assay (Figure 3B). Although we found a significant reduction in EAEC strain 042 binding to HEp-2 cells treated with FN siRNA as compared to cells treated with scrambled siRNA (Figure 3C), no difference in IL-8 secretion was observed (Figure 3D).

### Involvement of AAF/II fimbriae in FN-mediated IL-8 secretion

**Figure 4:**
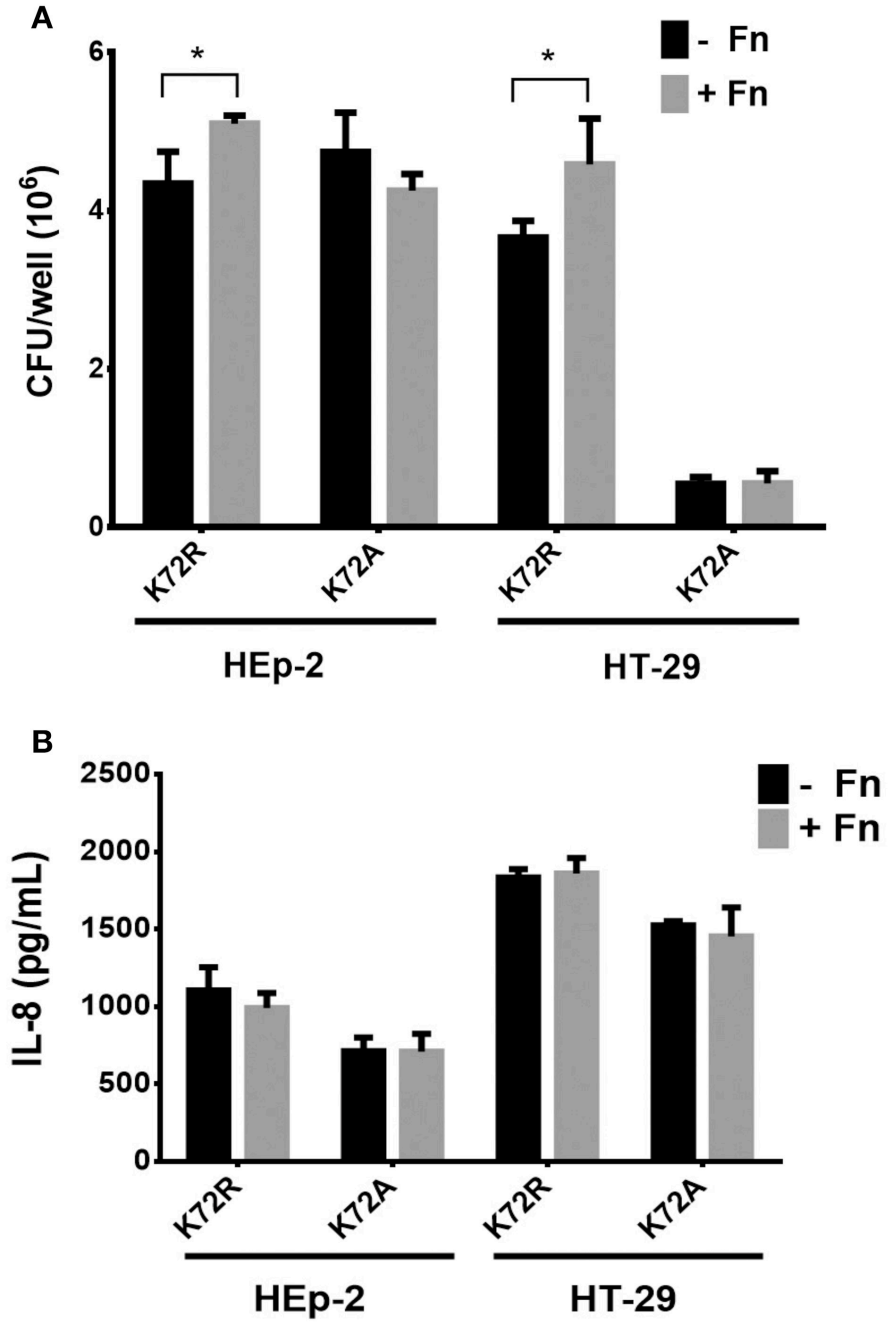
**Involvement of AAF/II in fibronectin-mediated IL-8 secretion. (A)** The number of bacteria adherent to HEp-2 or HT-29 cells pre-incubated with 7.5 μg/mL of fibronectin (+Fn) or media only (−Fn) and then infected with K72R or K72A strains. **(B)** Levels of secreted IL-8 at 3 h post infection. Bars represent the mean of three experiments, with the error bars indicating one standard deviation. ^*^Denotes statistical significant difference (*P* < 0.05).

Previous reports demonstrated that the substitution of Lys72Arg (K72R) in AafA proteins did not affect FN binding, whereas the Lys72Ala (K72A) substitution yielded significant reduction in binding to this ECM protein (Berry et al., 2014). In order to evaluate whether FN/AAF fimbriae interaction plays a role in the inflammation induced by EAEC on epithelial cells, we infected HEp-2 and HT-29 cells with the EAEC 042*aafA* mutant strain transformed with a pBAD30 plasmid harboring the native *aafA* gene with a site-directed mutation in the Lys72 residue (K72A and K72R strains). As expected, this amino-acid substitution significantly increased the adherence of the AafA mutant K72R in the presence of FN to HEp-2 and HT-29 cells. On the contrary, there was no difference in the adherence of the AafA mutant K72A in either the presence or absence of FN (Figure 4A). In both cell models, there was no difference in the induction of IL-8 secretion in cells infected with the mutants tested (Figure 4B).

### Interaction of FN with AAF/II fimbriae on epithelial cells infected with EAEC

**Figure 5:**
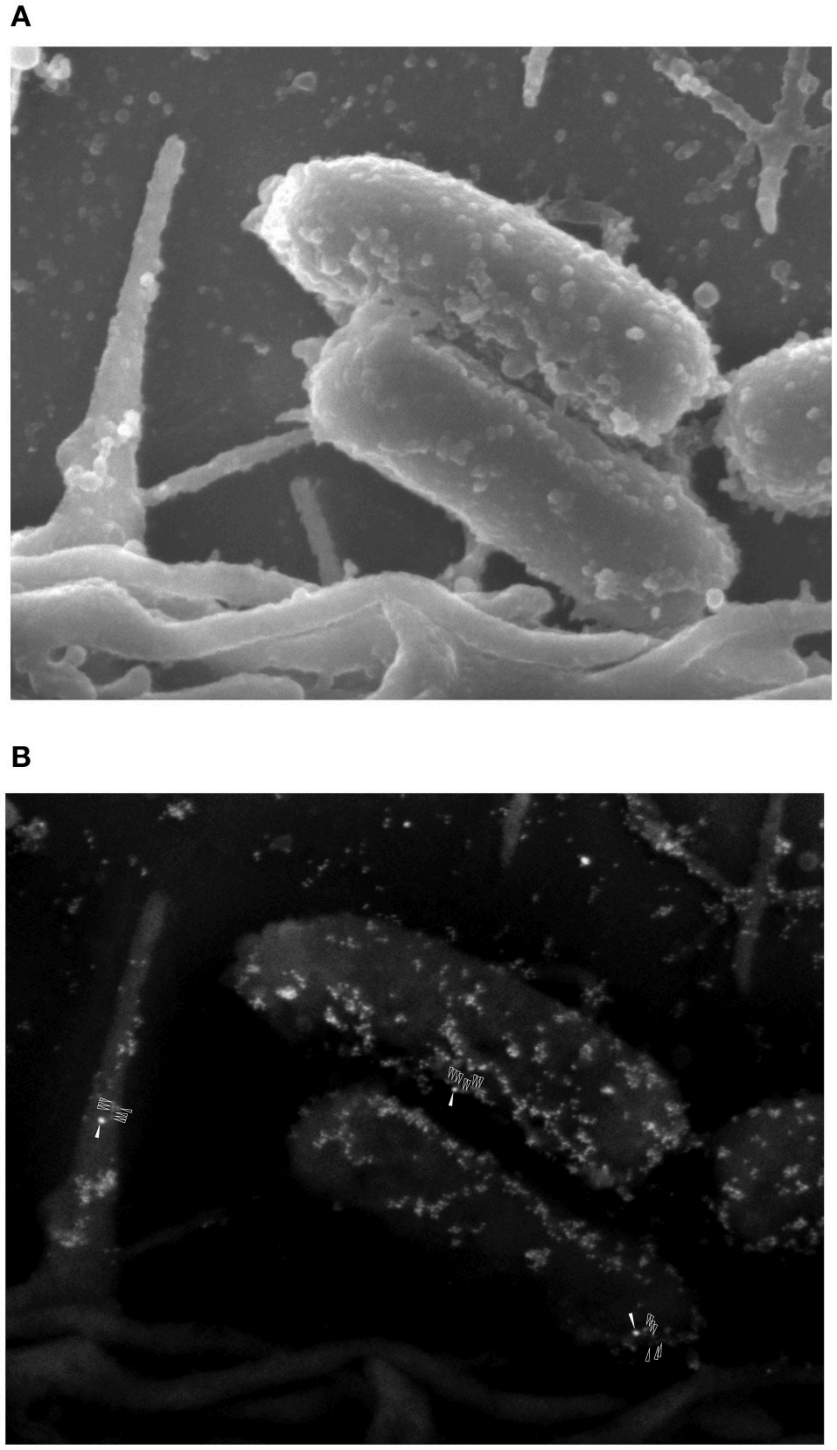
**Ultrastructural analysis of the interaction of fibronectin with AAF/II on EAEC-infected epithelial cells. (A)** An SEM image of EAEC 042 adhering to the surface of an HEp-2 cell. **(B)** An SEM image employing backscattered electron detection. The presence of fibronectin was evidenced with anti-fibronectin antibodies and anti-mouse IgG conjugated to 20-nm gold particles (solid white arrows). AAF/II was immunostained with rabbit anti-AafA serum plus anti-rabbit IgG conjugated to 10-nm gold particles (unfilled white arrows).

Our data suggests that FN increased adherence of EAEC to cells, but this increase was not related to high levels of *IL8* gene expression or IL-8 secretion. To understand these findings, we performed immunogold SEM assays on HEp-2 cell monolayers infected with EAEC in the presence of FN. This ultrastructural analysis revealed compelling evidence of the formation of an AAF/II-FN complex observed at the surface of the bacterium, as well as on the epithelial cell surface (Figure 5). These findings confirm that FN is involved in EAEC adherence to the cell surface mediated by AAF/II fimbriae.

## Discussion

FN is an ECM protein involved in several biological processes and may be recognized as a receptor by many bacterial pathogens (Henderson et al., 2011). However, one of the main questions regarding the ability of pathogens to adhere to FN is the biological significance of this binding in the context of disease establishment. It has been recognized for some time that FN plays a role in EAEC 042's adherence to epithelial cells through interaction with AAF/II fimbriae, the major EAEC virulence factor known to be involved in both adherence and inflammation (Farfan et al., 2008; Konar et al., 2012; Izquierdo et al., 2014a). In this study, we focused on determining whether the AAF/II-FN interaction constitutes a signal that triggers the expression of genes encoding pro-inflammatory markers resulting in an increased inflammatory response in infected host epithelial cells. We found that the addition of soluble FN to cultured epithelial cells increased adhesion of EAEC 042 in both epithelial cell models, HEp-2 and HT-29 (Figure 1B), as expected according to previously published data (Farfan et al., 2008). However, no difference in IL-8 secretion was observed in these experiments (Figure 1C). IL-8 is a cytokine responsible for the chemoattraction and recruitment of polymorphonuclear neutrophils (PMNs) at the site of infection, and is a marker widely used to evaluate inflammation response to pathogens (Szabady and McCormick, 2013). A previous report showed that AAF fimbriae were required for EAEC-induced PMN transmigration *in vitro* (Boll et al., 2012). To characterize the role that FN is playing in EAEC-induced PMN transmigration, inverted intestinal cells were infected with EAEC 042 in the presence or absence of FN as described (Vergara et al., 2015). No differences in the migration of PMNs were found (data not shown). These observations might be explained by the fact that similar amounts of IL-8 were secreted by cells infected with EAEC in the presence or absence of FN (Figure 1C).

Diarrheagenic *E. coli* strains induce secretion of IL-8 by activation of kinase mitogen activated protein (MAPK) ERK-1/2, JNK, and p38MAPK, leading to the induction of the transcription factors NF-κB and AP-1 (Khan and Konar, 2010). Therefore, we investigated expression of the NF-κB pathway genes in cells infected with EAEC in the presence or absence of FN. Our analysis showed that NF-κB pathway gene expression is FN-independent, since no significant quantitative differences in transcription were found in infected cells in the presence or absence of FN. Surprisingly though, a four-fold decrease in the expression of the *IL8* gene was observed when FN was added, suggesting that perhaps FN impedes AAF/II from inducing IL-8 secretion (Figure 2A). However, no differences in the expression of the *IL8* gene were found at post-infection time points (Figure 2B). These observations suggested that FN is not involved in the expression of *IL8* gene induced by EAEC.

To further confirm or rebut these findings, we then sought to investigate the effect of attenuation of the FN-encoding gene on HEp-2 cells by utilizing specific siRNA. This approach reduced FN production. Quantitative adherence and IL-8 secretion experiments were then performed with FN-producing and FN-negative HEp-2 cells. While EAEC adherence was reduced, in agreement with previous data, the reduced amount of surface-associated FN on HEp-2 cells had no effect in the amount of secreted IL-8 found in the media of infected cells (Figure 3).

Based on these results, we evaluated the involvement of AAF/II in this process. An EAEC 042*aafA* mutant expressing recombinant AafA proteins with directed mutations Lys72Arg (K72R) and Lys72Ala (K72A) (Figure 4) were used. In the case of the K72R mutant, the strain behaved like the wild-type EAEC, as increased cell adherence was observed in the presence of FN. Yet again, IL-8 secretion remained unchanged between treatments. For the K72A strain, which is not capable of binding to FN (Berry et al., 2014), no change in cell adhesion or secretion of IL-8 was observed; demonstrating that FN participates in AAF/II-mediated adherence of EAEC to cells, but is not involved in the inflammatory IL-8 response characteristic of EAEC 042 in this model study.

Our results demonstrate the role of FN as a bridging molecule, connecting the bacterium with the epithelial cell surface. Furthermore, our data strongly suggest that FN-mediated binding is not necessary for the activation of intracellular signaling cascades involved in the inflammatory response against EAEC infection. SEM immunogold analysis clearly showed that AAF/II fimbriae bind to either FN or the cell surface (Figure 5). AAF/II fimbriae also have the ability to bind to other receptors, such as laminin, collagen IV, cytokeratin 8, epidermal growth factor receptor, Thrombospondin-1, or glucose-regulated protein (Farfan et al., 2008; Konar et al., 2012; Izquierdo et al., 2014b). Upcoming studies in our laboratory will evaluate the role of these proteins in the inflammation process, in order to decipher the pathways involved in the inflammatory response elicited by epithelial cells after EAEC infection.

Previous studies have associated the inflammatory response induced by EAEC with the AafB minor subunit of AAF/II, presumably a tip adhesin protein. IL-8 released from intestinal epithelial cells infected with the EAEC 042*aafB* mutant strain was reduced in comparison to the wild-type strain (Harrington et al., 2005). In contrast, the binding of EAEC to FN is mediated by the AafA major subunit of AAF/II, and the interaction between these two proteins involves electrostatic interactions attributed to the presence of basic residues in the AAF (Berry et al., 2014). Given our experimental data, we hypothesize that when EAEC reaches its target epithelial cells in the gut environment, AAF/II may bind to adherence-associated receptors, such as FN, which as others described above, are recognized by AafA, or pro-inflammatory receptors that activate intracellular signaling cascades involved in mounting an inflammatory response, which are recognized by AafB. In this context, the binding of EAEC to cellular FN through AafA increases the number of bacteria at the cell surface, leaving AafB unavailable to interact with pro-inflammatory receptors, thereby dampening the secretion of pro-inflammatory cytokines, such as IL-8. The identification of an AafB protein receptor would help clarify or support this hypothesis, as well as aid in further understanding as to the role of AAF/II in immunomodulation of the inflammatory response during EAEC infections.

In conclusion, we report that while the presence of FN favors the interaction of EAEC 042 with host cultured epithelial cells, this ECM protein is not involved in the inflammatory response induced by EAEC.

## Author contributions

DY carried out the infection assay, IL-8 measurement and interpretation of data; MI, carried out the siRNA experiments and participate in the interpretation of data, FR-P participated in the interpretation of data; JN participated in the interpretation of data; JG carried out the SEM analysis, interpretation of data and manuscript writing, RV participated in the design of the study; MF participated in the design of the study, acquisition of data, interpretation of data and manuscript writing and final approval of the manuscript.

### Conflict of interest statement

The authors declare that the research was conducted in the absence of any commercial or financial relationships that could be construed as a potential conflict of interest.
